# Changes in Biomarkers of Redox Status in Saliva of Pigs after an Experimental Sepsis Induction

**DOI:** 10.3390/antiox11071380

**Published:** 2022-07-16

**Authors:** María José López-Martínez, Damián Escribano, Alba Ortín-Bustillo, Lorena Franco-Martínez, Luis Guillermo González-Arostegui, José Joaquín Cerón, Camila Peres Rubio

**Affiliations:** 1 Interdisciplinary Laboratory of Clinical Analysis, Interlab-UMU, Regional Campus of International Excellence “Mare Nostrum”, University of Murcia, 30100 Murcia, Spain; mariajose.lopezm96@gmail.com (M.J.L.-M.); det20165@um.es (D.E.); alba.ortinb@um.es (A.O.-B.); lorena.franco2@um.es (L.F.-M.); luisgarostegui@gmail.com (L.G.G.-A.); jjceron@um.es (J.J.C.); 2 Department of Animal Production, Regional Campus of International Excellence “Mare Nostrum”, University of Murcia, 30100 Murcia, Spain; 3 Department of Animal and Food Science, School of Veterinary Science, Universitat Autònoma de Barcelona, 08193 Cerdanyola del Vallès, Spain

**Keywords:** antioxidants, cupric, ferric, inflammation, oxidative stress, peroxides

## Abstract

Saliva from pigs is gaining attention as an easy sample to obtain, being a source of biomarkers that can provide information on animal health and welfare. This study aimed to evaluate the changes that can occur in salivary biomarkers of the redox status of pigs with an experimentally induced sepsis. For that, the cupric reducing antioxidant capacity (CUPRAC), ferric reducing ability of saliva (FRAS), Trolox equivalent antioxidant capacity (TEAC), advanced oxidation protein products (AOPP), ferrous oxidation-xylenol orange (FOX), peroxide activity (POX-Act), and reactive oxygen-derived compounds (d-ROMs) were measured in the saliva of pigs with experimentally induced sepsis by endotoxin lipopolysaccharide (LPS), non-septic inflammation induced by turpentine, and in healthy individuals before and after 3 h, 6 h, 24 h, and 48 h. AOPP, POX-Act, and d-ROMs in the sepsis group were higher than in the control from 3 h to 24 h after the inoculation. CUPRAC, FRAS, and TEAC were higher in sepsis than the control group at 24 h. These changes were of higher magnitude than those that occurred in the turpentine group. In conclusion, our findings reveal that sepsis produces changes in salivary biomarkers of redox status, which opens the possibility of using them as potential biomarkers in this species.

## 1. Introduction

The use of saliva for the determination of biomarkers has gained special attention in the last decade due to its easy collection, being of particularly high interest in pigs, in which blood collection is highly stressful. In addition, it has been shown that the saliva matrix is appropriate for health monitoring and disease diagnosis [1,2].

Infectious diseases in pigs are possibly the most important health concern in swine production [3,4]. Depending on factors, such as the immune status of the pig, pathogen involved, and environment, the disease can progress to sepsis and death. In addition, the intensification and globalization of the swine industry are being accompanied by a global spread of pathogens at local and international scales, which, together with the need for appropriate use of antibiotics and the withdrawal in the European Union of zinc oxide supplementation, highlights the importance of the correct detection and control of sepsis in pigs [5,6].

The endotoxin lipopolysaccharide (LPS) challenge is a validated model of sepsis in pigs [7]. In addition, porcine models of LPS-induced sepsis have been developed to simulate human sepsis [8,9]. The LPS is an outer-membrane component of Gram-negative bacteria that provokes a systemic inflammatory response, which includes the activation of free-radical-generating molecules in various types of cells that initiate tissue damage [10,11].

Free-radical molecules and other oxidants are produced constantly as a result of the respiratory chain, and the antioxidant system works continually to maintain the oxidant levels under control and without damage to the cells [12]. The imbalance between both the oxidant and antioxidant system is involved in the etiology of various diseases. Several studies have already shown that biomarkers of oxidative status can be measured in the saliva of pigs [13] and other species, such as bovine [14], canine [15], ovine [16], as well as humans [17]. To investigate the effects of a natural antioxidant compound during human sepsis, the LPS-induced model in piglets was used and various redox biomarkers were evaluated in serum [18]. However, to the author’s knowledge, no information is available yet about the changes that occur in the oxidative status of the saliva of pigs during induced sepsis.

The objective of this study was to evaluate the changes that can occur in biomarkers of redox status in the saliva of pigs with sepsis. For this purpose, an experimental trial using LPS was made. In addition, an experimentally induced non-septic inflammation by turpentine administration was performed in order to compare sepsis with a non-septic inflammatory condition. In these trials, a profile, including four antioxidant (cupric reducing antioxidant capacity (CUPRAC), ferric reducing ability of saliva (FRAS), Trolox equivalent antioxidant capacity (TEAC) and uric acid) and four oxidant biomarkers (advanced oxidation protein products (AOPP), ferrous oxidation-xylenol orange (FOX), peroxide activity (POX-Act), and reactive-oxygen-derived compounds (d-ROMs)) was measured.

## 2. Materials and Methods

### 2.1. Animals, Housing, and Experimental Design

The experimental protocol was approved by the Ethical Committee on Animal Experimentation (CEEA) of the University of Murcia (CEEA 563/2019) according to the European Council Directives considering the protection of animals used for experimental purposes. In addition, this study complies with Animal Research: Reporting of In Vivo Experiments (ARRIVE) guidelines for the care and use of animals.

In total, 15 growing male pigs were included in this study. They were in the mid-fattening period and belonged to the Experimental Farm of the University of Murcia (Murcia, Spain). All of them had water and a balanced diet ad libitum and were housed with a minimum space of 0.65 m^2^ per animal (Council Directive 2001/88/CE of 23 October 2001 amending Directive 91/630/CEE concerning minimum standards for the protection of pigs) and a standard temperature of 24 ± 2 °C. During the study, the pigs were 14 weeks old and had a median weight of 51.5 kg (interquartile range 48–53 kg).

Before starting the experiment, the animals were adapted to the experimental conditions for a week and then randomized and divided into three groups. The first group (*n* = 5; control group) received saline treatment (2 mL) by intramuscular route. The second group (*n* = 5; LPS group) received a single dose of 30 ug/kg LPS from Escherichia Coli (LPS; O55:B5, Sigma-Aldrich) reconstituted in sterile saline solution by intramuscular injection [19,20]. In the third group (*n* = 5, TURP group), 8 mL of TURP (oil of turpentine purified, Sigma–Aldrich) was given by two 4 mL subcutaneous injections in each front flank per animal. All injections were completed between 8 and 9 am. In some of these saliva samples, procalcitonin was measured for a previous study [21].

### 2.2. Sampling Procedure

Saliva samples were obtained 24 h before (baseline) the saline, LPS, or TURP injections and at 3, 6, 24, and 48 h after. Saliva was collected using a polypropylene sponge clipped to a metal rod. The pigs were allowed to chew the sponge without forcing them. Then the sponges were placed in Salivette tubes (Sarstedt, Aktiengesellschaft & Co. D-51588 Nümbrecht, Germany) and centrifuged at 3000× *g* for 10 min. The saliva samples were collected and stored in Eppendorf tubes at −80 °C until analysis.

### 2.3. Assessment of Salivary Biomarkers of Oxidative Status

The salivary concentration of CUPRAC, FRAS, TEAC, uric acid, and AOPP was determined using automated techniques previously described and validated for the saliva of pigs [13].

The measurement of FOX was based on the oxidation of ferrous to ferric ions by lipid hydroperoxides in the sample as previously published [22] and results were expressed in µmol of peroxide hydrogen per L of the sample (µmol/L).

The POX-Act determination was based on the assay described by Tatzber et al. [23] in which the oxidation of 3,5,3′5′-Tetramethylbenzidine (TMB) by peroxides in the sample is monitored. The results were also expressed in µmol of peroxide hydrogen per L of the sample (µmol/L).

Salivary d-ROMs levels were determined based on monitoring the N,N-Diethyl-p-phenylenediamine radical cation concentration as previously described [24] with results expressed in Carratelli units (Carr units).

The assays were performed using the Olympus AU400 (AU400 Automatic Chemistry Analyser, Olympus Europe GmbH, Hamburg, Germany) and showed lower than 15% imprecision. More details about the assays are described in Table S1.

### 2.4. Statistical Analysis

Data (Spreadsheet S1) were analyzed using GraphPad Prism software Inc. (GraphPad Prism, version 8 for Windows, Graph Pad Software Inc., San Diego, CA, USA). The normality of the distribution of the results was assessed by using the D’Agostino and Pearson test, giving a nonparametric distribution; therefore, all data were normalized by logarithmic transformation (Y = Log[Y]) before analysis. A two-way analysis of variance (ANOVA) mixed model of repeated measures was performed, and time and treatment were used as random factors to account for multiple observations. Sidak’s multiple comparison test was used to compare the groups over time. Tukey’s multiple comparison test was applied to the data considering the sampling time as a repeated measure, with fixed effects of treatment. The effects were considered as significant if *p*-values were <0.05.

## 3. Results

### 3.1. Antioxidant Biomarkers

**Figure 1:**
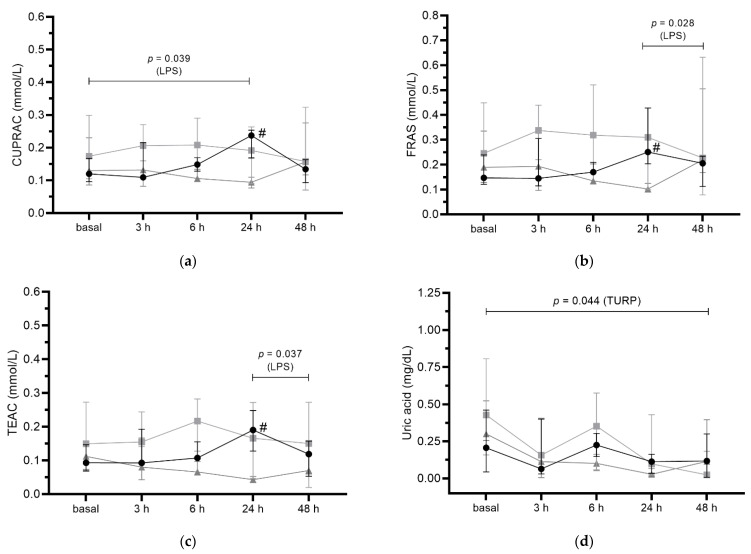
(**a**) Cupric reducing antioxidant capacity (CUPRAC), (**b**) ferric reducing ability of saliva (FRAS), (**c**) Trolox equivalent antioxidant capacity (TEAC), and (**d**) uric acid concentrations in control (▲), lipopolysaccharide (LPS)-treated pigs (●), and turpentine (TURP)-treated pigs (■) before (basal) and after 3 h, 6 h, 24 h, and 48 h the inoculations. The results are presented as median with an interquartile range. **#**, significantly different from the control group (*p* < 0.05; one-way ANOVA with Sidak’s multiple comparisons test). Differences between times are indicated by bars and the obtained *p*-value (one-way ANOVA with Tukey’s multiple comparison test).

LPS-treated pigs had significant increased concentrations of CUPRAC at 24 h when compared to basal time (*p* = 0.039, Figure 1a). TEAC showed a tendency to increase (*p* = 0.056, Figure 1b) at 24 h in comparison to basal time in this group. When compared to healthy controls, the LPS group showed significantly higher concentrations of CUPRAC (*p* = 0.004; Figure 1a), FRAS (*p* = 0.028; Figure 1b), and TEAC (*p* = 0.001; Figure 1c) 24 h after injection. 

TURP-treated pigs showed no changes in antioxidant biomarkers throughout the study, except for uric acid (Figure 1d), which was decreased at 48 h in relation to baseline (*p* = 0.044). There was no significant difference comparing control and TURP-treated pigs at any time point (*p* > 0.05) (Figure 1).

### 3.2. Oxidant Biomarkers

**Figure 2:**
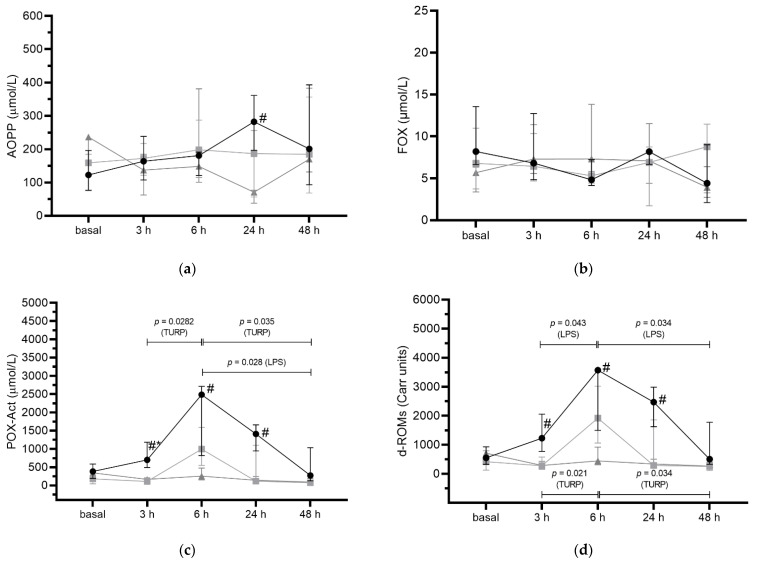
Salivary (**a**) advanced oxidation protein products (AOPP), (**b**) ferrous oxidation-xylenol orange (FOX), (**c**) peroxide activity (POX-Act), and (**d**) reactive-oxygen-derived compounds (d-ROMs) concentrations in control (▲), lipopolysaccharide (LPS)-treated pigs (●), and turpentine (TURP)-treated pigs (■) before (basal) and after 3 h, 6 h, 24 h, and 48 h the inoculations. The results are presented as median with an interquartile range. **#**, significantly different from the control group; *, significantly different from TURP-treated pigs (*p* < 0.05; one-way ANOVA with Sidak’s multiple comparisons test). Differences between times are indicated by bars and the obtained *p*-value (one-way ANOVA with Tukey’s multiple comparison test).

LPS-treated pigs presented significantly higher POX-Act (Figure 2c) and d-ROMs (Figure 2d) than control at 3 h (*p* < 0.039), 6 h (*p* < 0.033) and 24 h (*p* < 0.001), reaching the highest values at 6 h. They also showed higher POX-Act than TURP-treated pigs at 3 h (*p* < 0.036). When compared to control, the LPS group showed significantly higher concentrations of AOPP (*p* = 0.004, Figure 2a) 24 h after the injections.

There was no significant difference in FOX concentrations between groups and between time points (*p* > 0.05) (Figure 2b).

## 4. Discussion

To the best of our knowledge, this is the first study that describes changes in biomarkers of redox status in pigs with sepsis. The pigs in our study showed, after LPS administration, increased body temperature as well as increased white blood cell count and C-reactive protein (Table S2), and the presence of symptoms, such as depression, lethargy, and increased respiratory rate; which proved the validity of LPS administration as a model for sepsis in pigs, as has been previously described [21,25].

The pigs with sepsis showed higher values in three of the oxidants measured in our study (AOPP, POX-Act, and d-ROMs) compared with the healthy control pigs. POX-Act and d-ROMs presented earlier increases, being significantly higher at 3 h after the induction of sepsis, whereas AOPP showed significant increases at 24 h. Although no studies about salivary oxidative biomarkers in pigs with sepsis have been previously reported, pigs with sepsis showed increased 8-Iso-prostaglandin F2α, a lipid peroxidation product, in plasma [26,27]. Furthermore, in piglets with sepsis induced by LPS, liver and lung content of two oxidants, such as malondialdehyde and protein carbonyl, were increased compared to the control animals [18]. In addition, an increment in oxidants in serum was also observed in human sepsis [28,29]. Therefore, it can be postulated that sepsis induces an increase in oxidants in the organism that can be reflected in saliva.

The increases in oxidant biomarkers in sepsis could be due to the activation of the phagocytic NADPH oxidase complex, leading to the production of reactive nitrogen and oxygen species (RNS and ROS, respectively), and their sustained and excessive production cause damage to endothelial cells and organ failure, which is associated with increased morbidity and mortality in sepsis patients [30]. In our study, the concentrations of oxidants at 48 h in the septic group were similar to the basal and healthy group values, which indicates that production of ROS was not sustained, and that could be associated with the recovery experimented by the pigs in our conditions.

In our report, pigs with induced sepsis showed higher concentrations of CUPRAC, FRAS, and TEAC 24 h after LPS administration compared to the healthy ones. This is in line with previous results in humans, in which septic neonates presented higher levels of antioxidant enzymes in serum, such as superoxide dismutase and glutathione peroxidase [31]. In sepsis, different antioxidant responses can be obtained depending on the severity and outcome [29]. Patients who developed organ dysfunction and did not overcome sepsis showed decreased antioxidants, while those who survived presented increases over time, in line with our findings [32,33]. Therefore, it would be of interest to explore the potential of these biomarkers for predicting the outcome of sepsis.

The increased antioxidant response might be an attempt to counteract the damage that could be caused by the overproduced oxidants observed in this group. Namely, α-tocopherol and ascorbic acid are the first lines of defense against intravascular oxidants involved in sepsis [34]; this could explain our results, since CUPRAC, TEAC and FRAS measure both antioxidants.

In general, our findings reveal an increase in oxidant and antioxidant markers during sepsis in pigs, indicating that salivary biomarkers of redox status change in pigs with this condition. On the other hand, the animals that had local inflammation did not show the differences in redox biomarkers that occurred in the sepsis group. This will indicate that in our experimental conditions, the changes in redox biomarkers produced in sepsis are of a higher magnitude than those of non-septic inflammatory conditions. Further studies should be undertaken in saliva to elucidate if biomarkers of redox status could help to differentiate between septic and non-septic inflammation.

The small number of animals included in this study should be mentioned as a limitation; therefore, this should be considered a pilot study and the results need to be confirmed in a larger population. In addition, it would have been interesting to evaluate the possible correlation of the biomarkers concentrations between saliva and serum, although previous reports in pigs did not find a major correlation in these these analytes between both fluids [35]. Furthermore, the results obtained in this study were not corrected by protein concentration, in line with other studies performed with other biomarkers of oxidative stress in which this procedure was not recommended [36]. However, further research needs to be performed in the future to identify the necessity or not of the correction by protein when biomarkers of oxidative status are measured in animals’ saliva.

## 5. Conclusions

The saliva of pigs during experimental sepsis showed higher levels of the oxidant biomarkers AOPP, POX-Act, and d-ROMs compared to healthy animals. In addition, higher levels of the antioxidant markers CUPRAC, TEAC, and FRAS were also observed in pigs with sepsis. These findings could reflect that there is an increase in oxidants during sepsis in pigs and the mobilization of some antioxidants in order to protect against damage. Overall, this study shows that sepsis can induce changes in pigs’ saliva and opens the possibility of using the salivary biomarkers of redox status as potential biomarkers of sepsis in this species.

## Data Availability

The data is contained within this article and supplementary files.

## Supplementary Materials

The following supporting information can be downloaded at: https://www.mdpi.com/article/10.3390/antiox11071380/s1, Table S1: Description of the basis and reagents of each assay performed in the study; Table S2: Rectal temperature, white blood cell count (WBC), and C-reactive protein (CRP) of each animal included in the study before and 3 and 24 h after injection of lipopolysaccharide (LPS), turpentine (TURP), and saline (control group). Data of LPS and TURP groups were previously described [25]; Spreadsheet S1: Raw data.
