# Supplementary material for: Changes in Biomarkers of Redox Status in Saliva of Pigs after an Experimental Sepsis Induction
Source: Antioxidants (Basel). 2022 Jul 16;11(7):1380. doi: 10.3390/antiox11071380 (PMC9311702; doi:10.3390/antiox11071380)
Supplement: Supplementary file 1 [file antioxidants-11-01380-s001.zip › antioxidants-1789051-supplementary.pdf]

Table S1: Description of the basis and reagents of each assay performed in the study.

Method*	Basis of the method	Reagents (in home-made assays) or manufacture	
		**Reagent 1**	**Reagent 2**
**CUPRAC**	Reduction of Cu2+ to Cu1+ by the antioxidants present in the sample	0.25mM of bathocuproinedisulfonic acid disodium salt in phosphate buffer	0.5mM of Cu(II) sulphate anhydrous in ultrapure water
**FRAS**	Reduction of Fe3+ to Fe2+ by the antioxidants present in the sample	0.7mM of tripyridyltriazine, and 1.5mM of ferric chloride hexahydrate in acetate buffer	none
**TEAC**	Reduction of 2,2′-azino- bis(3-ethylbenzthiazoline-6-sulfonic acid) (ABTS) radical to ABTS by the antioxidants present in the sample	2mM of ABTS, 0.25μM of horseradish peroxidase (HRP), and 40μM of H2O2 in phosphate buffer	none
**Uric acid**	Oxidation of uric acid by uricase to produce allantoin and H2O2, which reacts with 4-aminoantipyrine and 3,5-dichloro-2-hydroxybenzene sulfonate	Beckman Coulter[a]	
**AOPP**	Oxidatively-modified albumin absorb at 340 nm in the presence of potassium iodide (KI) in acidic conditions	0.059M of KI in ultrapure water	Acetic acid, 50% (v/v)
**FOX**	Oxidation of Fe2+ to Fe3+ in acidic solution by oxidants in the sample	120μM of xylenol orange in 40mM H2SO4 with 1.37M glycerol and 20mM formic acid	150μM of iron D-gluconate dehydrate in 40mM H2SO4 with 1.37M glycerol and 20mM formic acid
**POX-Act**	Estimation of total peroxides through a peroxide–peroxidase reaction using tetramethylbenzidine (TMB) as the chromogenic substrate	0.7mM of TMB and 25mU of HRP in acetate buffer	none
**d-ROMs**	Estimation of total peroxides in the sample by monitoring the N,N-dyethylparaphenyldiamine (DEPPD) radical cation concentration based on Fenton's reaction	Acetate buffer	0.37M of DEPPD in ultrapure water

[a]Beckman Coulter Inc, Fullerton, CA, USA

*All assays were conducted in the Olympus AU400, at a constant temperature of 37°C and in a reaction time between 5 and 8 minutes.

Table S2: Rectal temperature, white blood cell count (WBC) and C-reactive protein (CRP) of each animal included in the study before, and 3 and 24h after injection of lipopolysaccharide (LPS), turpentine (TURP), and saline (control group). Data of LPS and TURP groups have been previously described [25].

Group	Pig ID	Sampling time	Rectal temperature (ºC)	WBC (x10^3 cells/µL)	CRP (µg/mL)
**LPS**	1	Basal	39.2	17.6	2.8
		T3	40.9	4.33	47.5
		T24	38.6	19.3	79.2
	2	Basal	39.1	10.2	5.6
		T3	40.7	7.32	11.4
		T24	38.9	18.6	54.8
	3	Basal	39.6	15.4	14.2
		T3	40.7	3.50	85.3
		T24	38.3	37.0	78
	4	Basal	39.4	12.8	4.6
		T3	40.4	2.85	43.8
		T24	38.4	17.4	64.6
	5	Basal	39.3	12.5	2.5
		T3	41.9	3.80	44.6
		T24	40.4	18.6	103.8
**TURP**	6	Basal	39.2	14.0	5.5
		T3	39.5	19.10	15.8
		T24	39.8	20.0	112.1
	7	Basal	39.5	15.2	1.1
		T3	39.6	18.57	16.4
		T24	39.4	17.1	115.7
	8	Basal	39.1	13.0	1.1
		T3	39.7	10.30	6.9
		T24	39.3	9.1	104.7
	9	Basal	39.2	17.8	4.8
		T3	39.5	22.09	19.9
		T24	38.3	17.4	91.9
	10	Basal	39.4	15.6	1.1
		T3	39.9	23.19	12.4
		T24	39	19.7	112.1
**CONTROL**	11	Basal	39	16.32	1.1
		T3	39.5	18.30	4.4
		T24	38.4	18.47	7.4
	12	Basal	39.3	12.31	3.97
		T3	39.5	16.67	10.6
		T24	38.4	13.95	11.7
	13	Basal	38.7	17.30	1.1
		T3	39.2	19.15	4
		T24	38.9	18.14	13.9
	14	Basal	38.5	18.26	1.1
		T3	39.4	18.90	10.9
		T24	39.5	15.60	10.6
	15	Basal	39.1	18.75	1.1
		T3	39	18.45	3.5
		T24	38.5	17.34	9.8
